# Supplementary material for: A bifunctional RNase H protein supports DNA replication and transcription in nucleus-forming Chimalliviridae phages
Source: bioRxiv. 2026 Oct 2:2026.10.01.755736. Preprint. [Version 1] doi: 10.64898/2026.10.01.755736 (PMC13648698; doi:10.64898/2026.10.01.755736)
Supplement: Supplementary file 1 [file NIHPP2026.10.01.755736V1-supplement-1.pdf]

bioRxiv preprint doi: https://doi.org/10.64898/2026.10.01.755736; this version posted October 2, 2026. The copyright holder for this preprint (which was not certified by peer review) is the author/funder, who has granted bioRxiv a license to display the preprint in perpetuity. It is made available under a CC-BY 4.0 International license.

SUPPLEMENTAL FIGURES

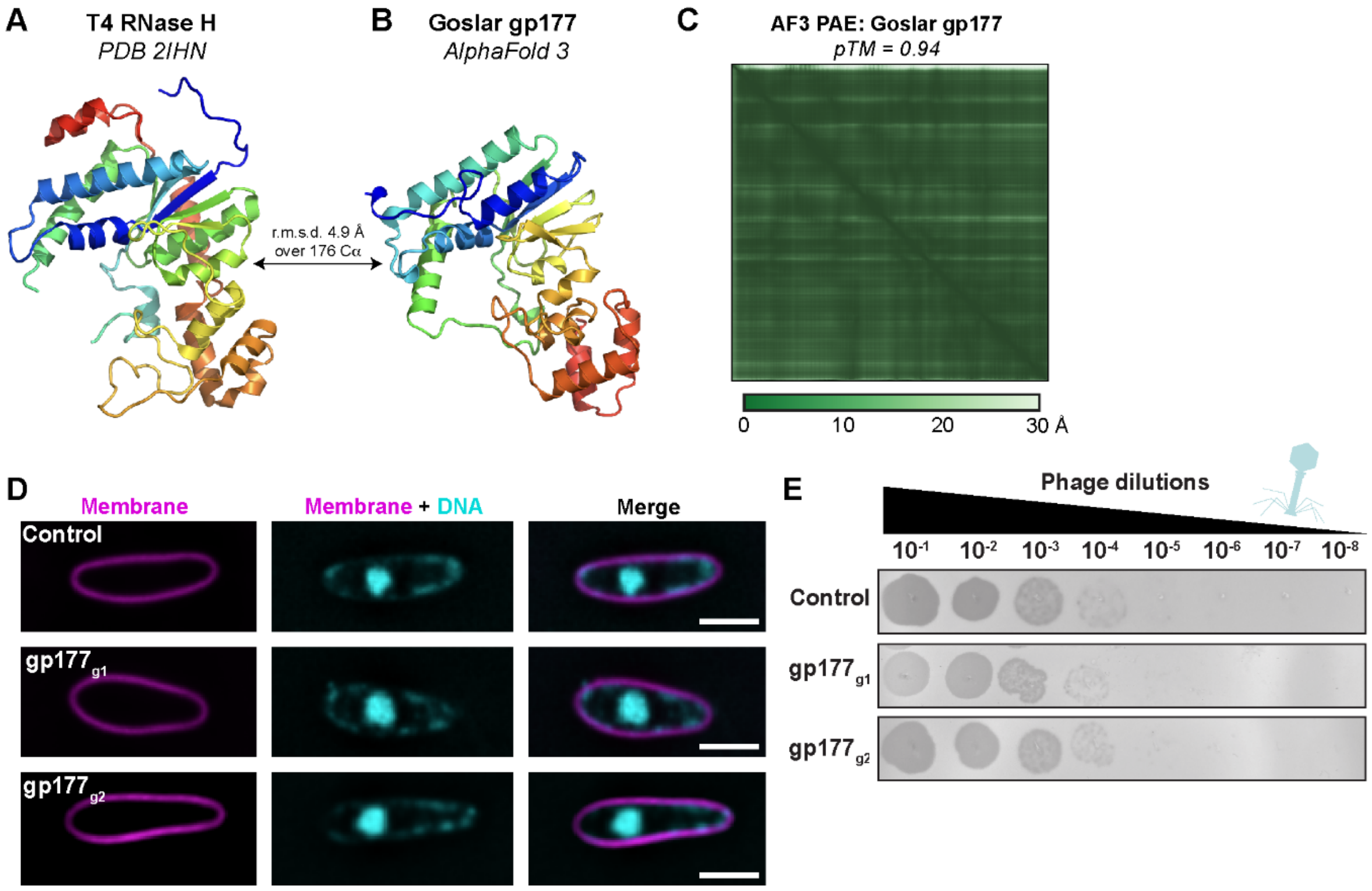

Figure S1. gp177 is dispensable for Goslar phage replication. (A) Crystal structure of T4 phage RNase H (PDB 2IHN) [63], colored as a rainbow from N-terminus (blue) to C-terminus (red). **(B)** AlphaFold 3 predicted structure of Goslar gp177, colored as a rainbow from N-terminus (blue) to C-terminus (red). **(C)** AlphaFold 3 predicted aligned error (PAE) plot for the structure prediction shown in panel (B). **(D)** Fluorescence microscopy analysis of non-targetting control (NT control) and gp177 knockdown infections. Cells were stained with FM4-64 to label membranes (magenta) and DAPI to visualize DNA (cyan). Scale bars, 2 μm. **(E)** Spot titer assays comparing phage progeny production in non-targeting control (NT) and gp177 knockdown strains using two independent guide RNAs (g1 and g2). The indicated serial dilutions of Goslar were spotted onto *E. coli* lawns.

bioRxiv preprint doi: https://doi.org/10.64898/2026.10.01.755736; this version posted October 2, 2026. The copyright holder for this preprint (which was not certified by peer review) is the author/funder, who has granted bioRxiv a license to display the preprint in perpetuity. It is made available under a CC-BY 4.0 International license.

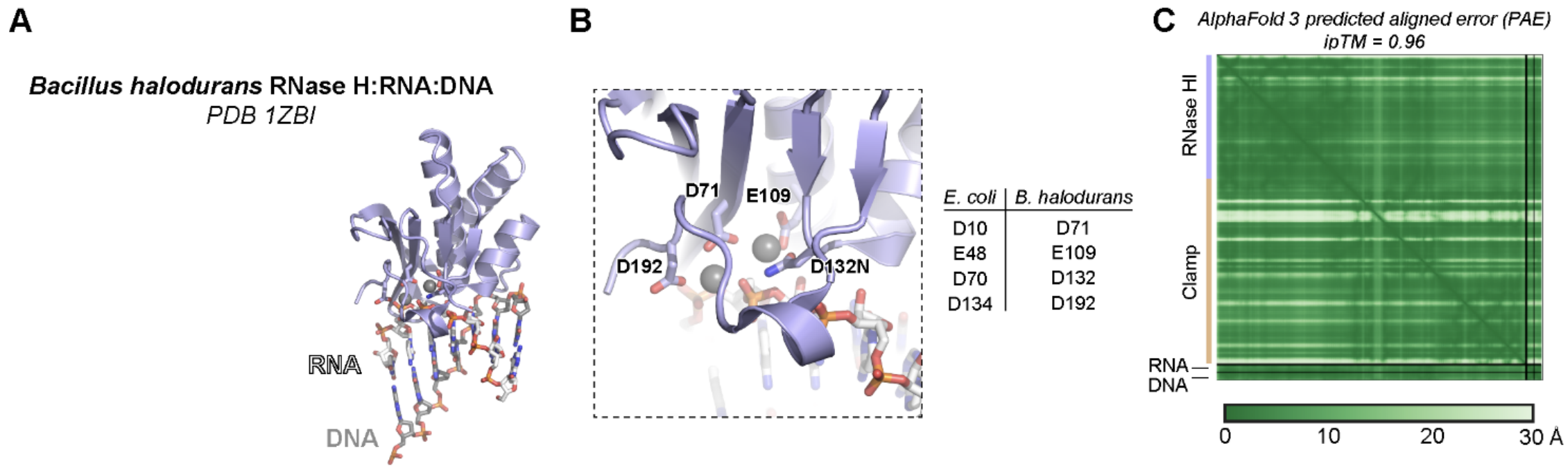

Figure S2. Rhc has a predicted RNase HI domain (A) Structure of *B. halodurans* RNase H bound to an RNA-DNA hybrid (PDB ID 1ZBI) [64]. **(B)** Closeup of the *B. halodurans* RNase H catalytic site, with conserved catalytic residues shown as sticks and two Mg^{2+} ions shown as spheres. *Right:* Equivalent active site residues of *E. coli* RNase H and *B. halodurans* RNase H. **(C)** AlphaFold 3 predicted aligned error (PAE) plot for the prediction shown in panel (

bioRxiv preprint doi: https://doi.org/10.64898/2026.10.01.755736; this version posted October 2, 2026. The copyright holder for this preprint (which was not certified by peer review) is the author/funder, who has granted bioRxiv a license to display the preprint in perpetuity. It is made available under a CC-BY 4.0 International license.

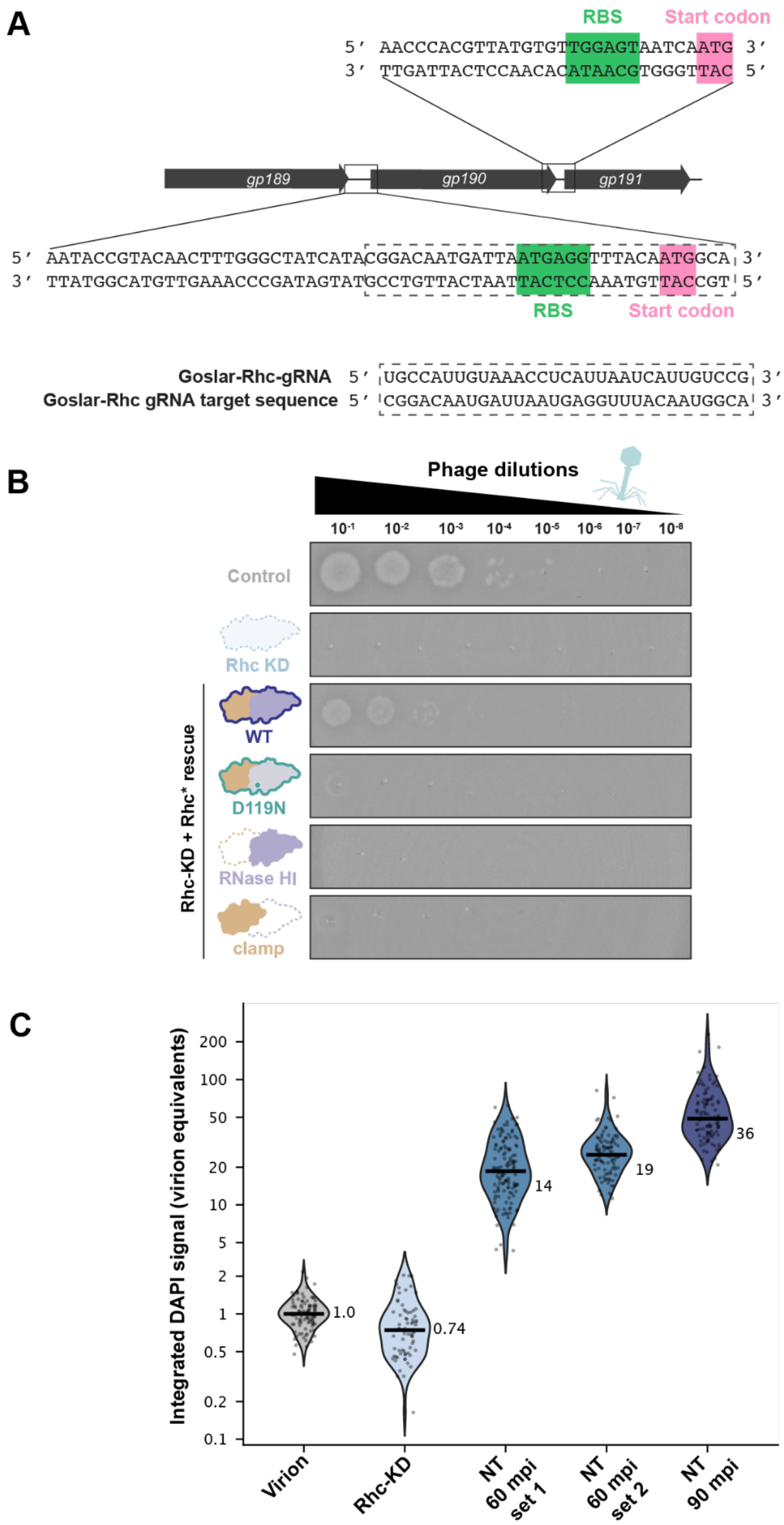

Figure S3. Goslar Rhc CRISPRi-ART knockdown strategy and phenotype (A) Schematic showing the genomic context of Goslar *gp190* (*Rhc*) and CRISPRi-ART guide RNA. Expanded views highlight the *gp190* 5′ untranslated and coding regions, indicating the likely ribosome-binding site (RBS; green) and start codon (magenta). The CRISPRi-ART guide RNA (gp190_gRNA) was designed to target the translation initiation region. **(B)** Representative spot titer assay of infectious phage production under gp190/Rhc knockdown and complementation by wild type (WT) or catalytic dead (D119N) CRISPRi-ART-resistant Rhc (Rhc*), or the isolated RNase H or clamp domains. Goslar lysates were pre-diluted 1:400, followed by 10-fold serial dilution and spotting on bacterial lawns. Dilution labels indicate the serial dilution factor applied after the initial 1:400 pre-dilution. **(C)** Preliminary analysis of DAPI signal per nucleus, expressed in virion equivalents (integrated DAPI signal relative to the median of free virions; n = 99 virions, 72 Rhc-KD nuclei at 60 mpi, and non-targeting control (NT) nuclei: 133 (set 1) and 100 (set 2) at 60 mpi and 101 at 90 mpi). Violins show kernel density estimates of log10(value), bars show medians, and dots show individual objects.

bioRxiv preprint doi: https://doi.org/10.64898/2026.10.01.755736; this version posted October 2, 2026. The copyright holder for this preprint (which was not certified by peer review) is the author/funder, who has granted bioRxiv a license to display the preprint in perpetuity. It is made available under a CC-BY 4.0 International license.

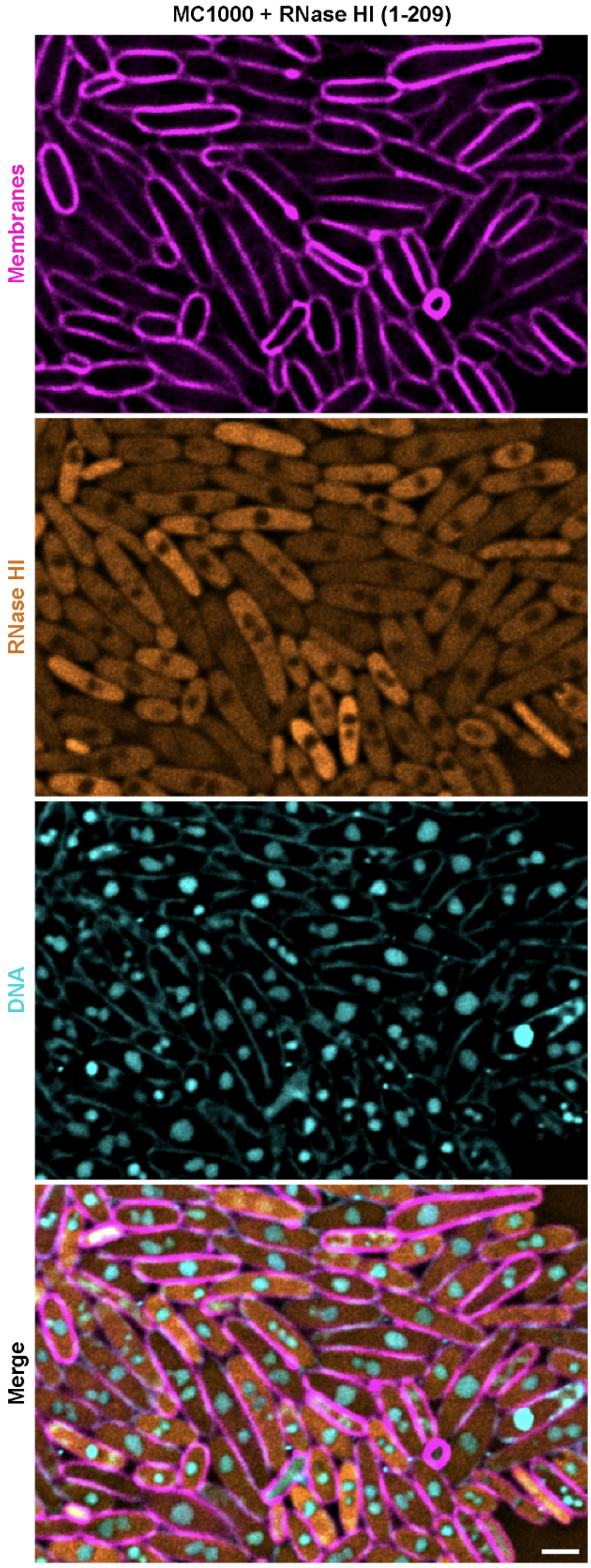

Figure S4. Localization of GFP-Rhc* RNase HI domain Goslar-infected *E. coli* MC1000. Representative fluorescence images of *E. coli* MC1000 expressing GFP-Rhc* RNase HI domain (residues 1-209). Cell membranes were labeled with FM4-64 (magenta), GFP-Rhc* is shown in orange, and DNA was stained with DAPI (cyan). Scale bar = 2 µm.

bioRxiv preprint doi: https://doi.org/10.64898/2026.10.01.755736; this version posted October 2, 2026. The copyright holder for this preprint (which was not certified by peer review) is the author/funder, who has granted bioRxiv a license to display the preprint in perpetuity. It is made available under a CC-BY 4.0 International license.

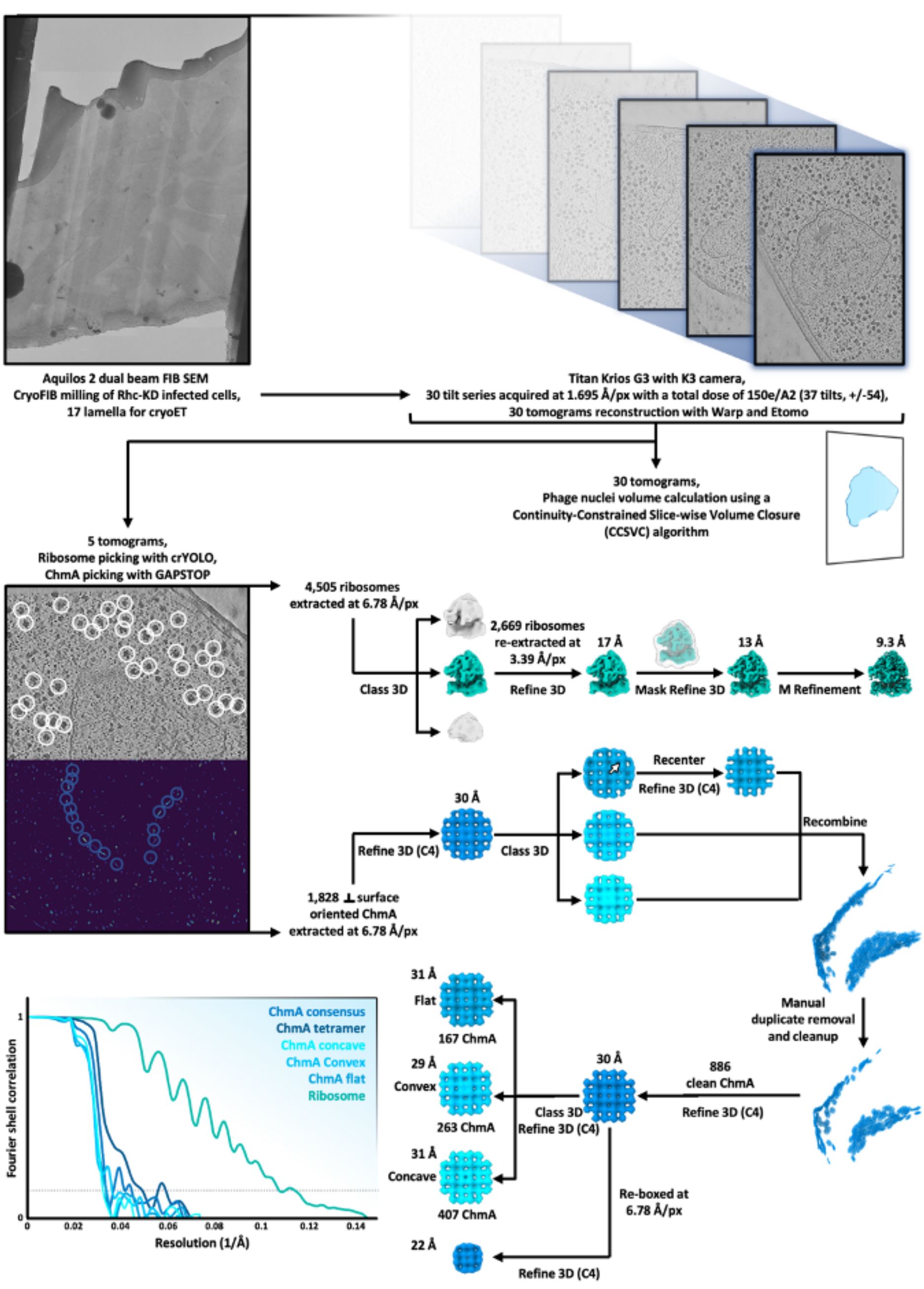

Figure S5. *In situ* cryoET data acquisition and subtomogram analysis of bacterial ribosomes and phage chmA in Goslar-infected Rhc-KD cells. Rhc-KD cells were cryoFIB-milled 60 min after Goslar infection, yielding 17 lamellae from which 30 tilt series (±54°, 150 $e^-/Å^2$) were acquired on a Titan Krios G3 equipped with a K3 detector. Tomograms were reconstructed using Warp and Etomo, and phage nuclei volumes were quantified using the CCSVC algorithm. Ribosomes and chmA were picked from five tomograms using crYOLO and GAPSTOP, respectively. Ribosome subtomogram analysis yielded a final resolution of 9.3 Å following refinement in the Relion/Warp/M pipeline. ChmA tetramers were refined with C4 symmetry and 3D classified to identify picking outliers. Duplicates and orientation outliers were then manually removed or corrected. Subsequent additional 3D classification of the clean dataset and focused 3D refinement resolved flat, convex, and concave shell conformations at 31-22 Å resolution, based on the FSC 0.143 criterion (grey dotted line).

bioRxiv preprint doi: https://doi.org/10.64898/2026.10.01.755736; this version posted October 2, 2026. The copyright holder for this preprint (which was not certified by peer review) is the author/funder, who has granted bioRxiv a license to display the preprint in perpetuity. It is made available under a CC-BY 4.0 International license.

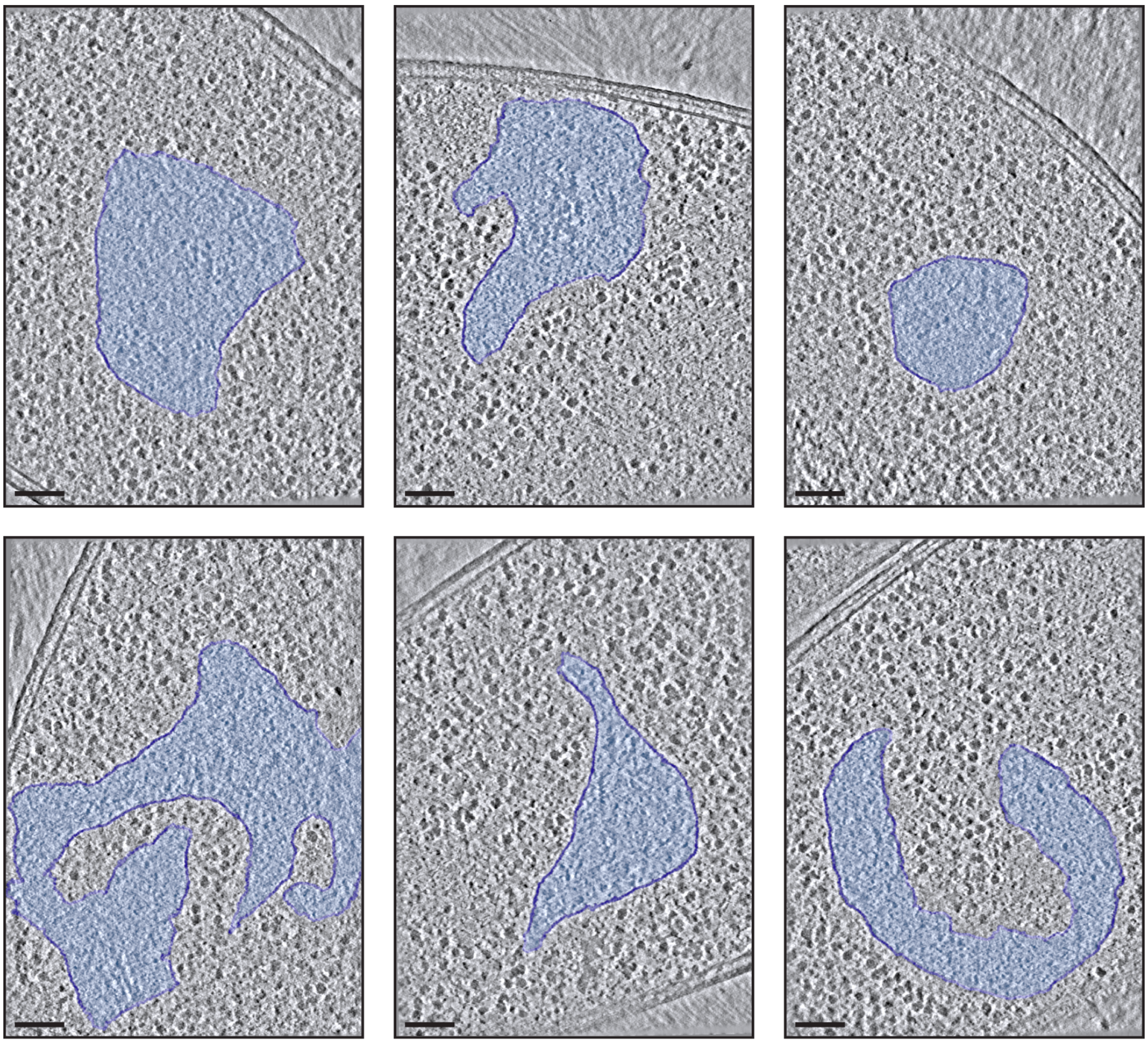

Figure S6. *In situ* cryoET of Rhc KD phage nuclei. Tomographic slices of cryoFIB milled *E. coli* cells show the different shapes of the nucleus 60 min after phage infection. Scale bar = 0.1 μm.

bioRxiv preprint doi: https://doi.org/10.64898/2026.10.01.755736; this version posted October 2, 2026. The copyright holder for this preprint (which was not certified by peer review) is the author/funder, who has granted bioRxiv a license to display the preprint in perpetuity. It is made available under a CC-BY 4.0 International license.

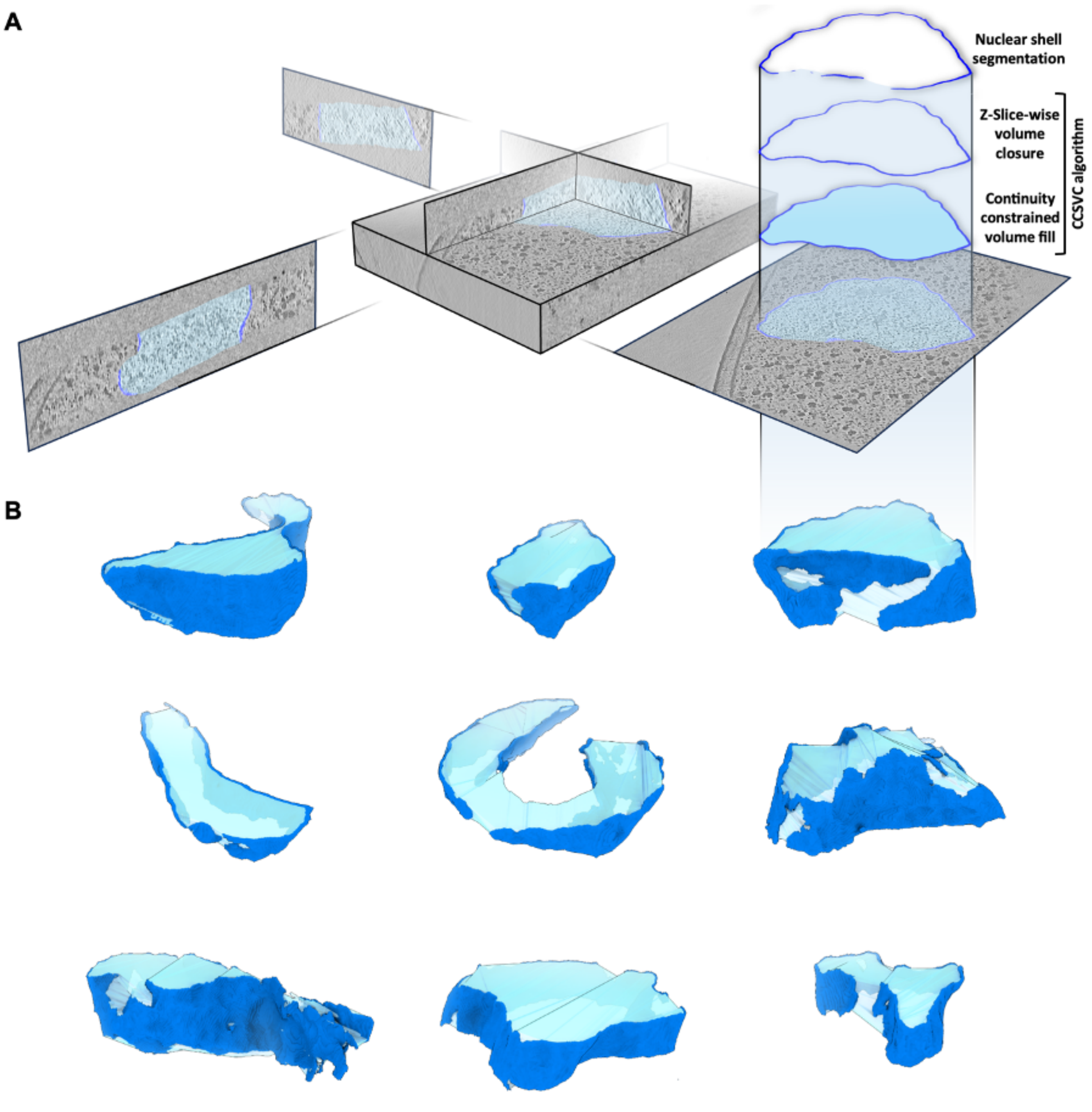

Figure S7. Phage nuclei volume calculation using the Continuity-Constrained, Slice-wise Volume Closure (CCSVC) algorithm. (A) Reconstructed tomograms of Rhc-KD cells were segmented to obtain a cryoEM density of the embedded phage nuclei. The segmentation was then converted into a binary mask and skeletonized to define endpoints for each z-slice. Endpoint pairs were connected by straight voxel paths to close boundaries in the preliminary fragmented segmentation of the phage nucleus. A closed binary mask allowed for subsequent filling of the cross-sectional area in each z-slice. **(B)** The CCSVC algorithm allowed for the generation of a three-dimensional binary volume enclosed by the phage nuclei. Generated volumes (transparent cyan) with their surrounding fragmented nuclear shell segmentation (blue) are shown for a subset of tomograms.

bioRxiv preprint doi: https://doi.org/10.64898/2026.10.01.755736; this version posted October 2, 2026. The copyright holder for this preprint (which was not certified by peer review) is the author/funder, who has granted bioRxiv a license to display the preprint in perpetuity. It is made available under a CC-BY 4.0 International license.

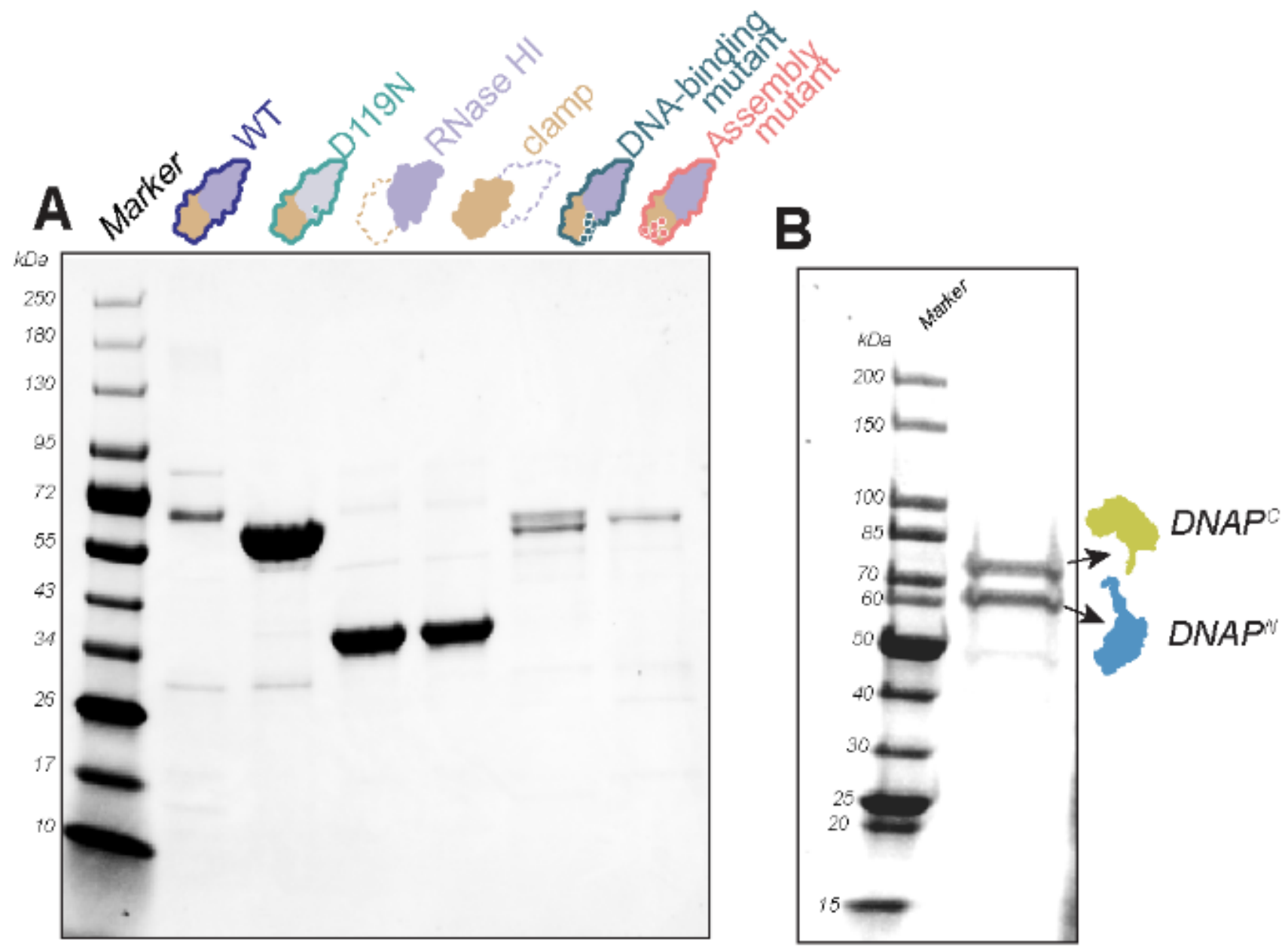

Figure S8. Purification of Rhc variants and the Goslar DNA polymerase complex. (A) Coomassie-stained SDS-PAGE showing purified WT, the catalytic-dead D119N mutant, the isolated RNase HI domain, the isolated clamp domain, the DNA-binding mutant, and the assembly mutant. Molecular-weight markers are indicated in kDa. **(B)** Coomassie-stained SDS-PAGE of the purified Goslar DNAP complex containing DNAP-C and DNAP-N. The positions of $DNAP^C$ and $DNAP^N$ are indicated by arrows. Molecular-weight markers are shown in kDa.

bioRxiv preprint doi: https://doi.org/10.64898/2026.10.01.755736; this version posted October 2, 2026. The copyright holder for this preprint (which was not certified by peer review) is the author/funder, who has granted bioRxiv a license to display the preprint in perpetuity. It is made available under a CC-BY 4.0 International license.

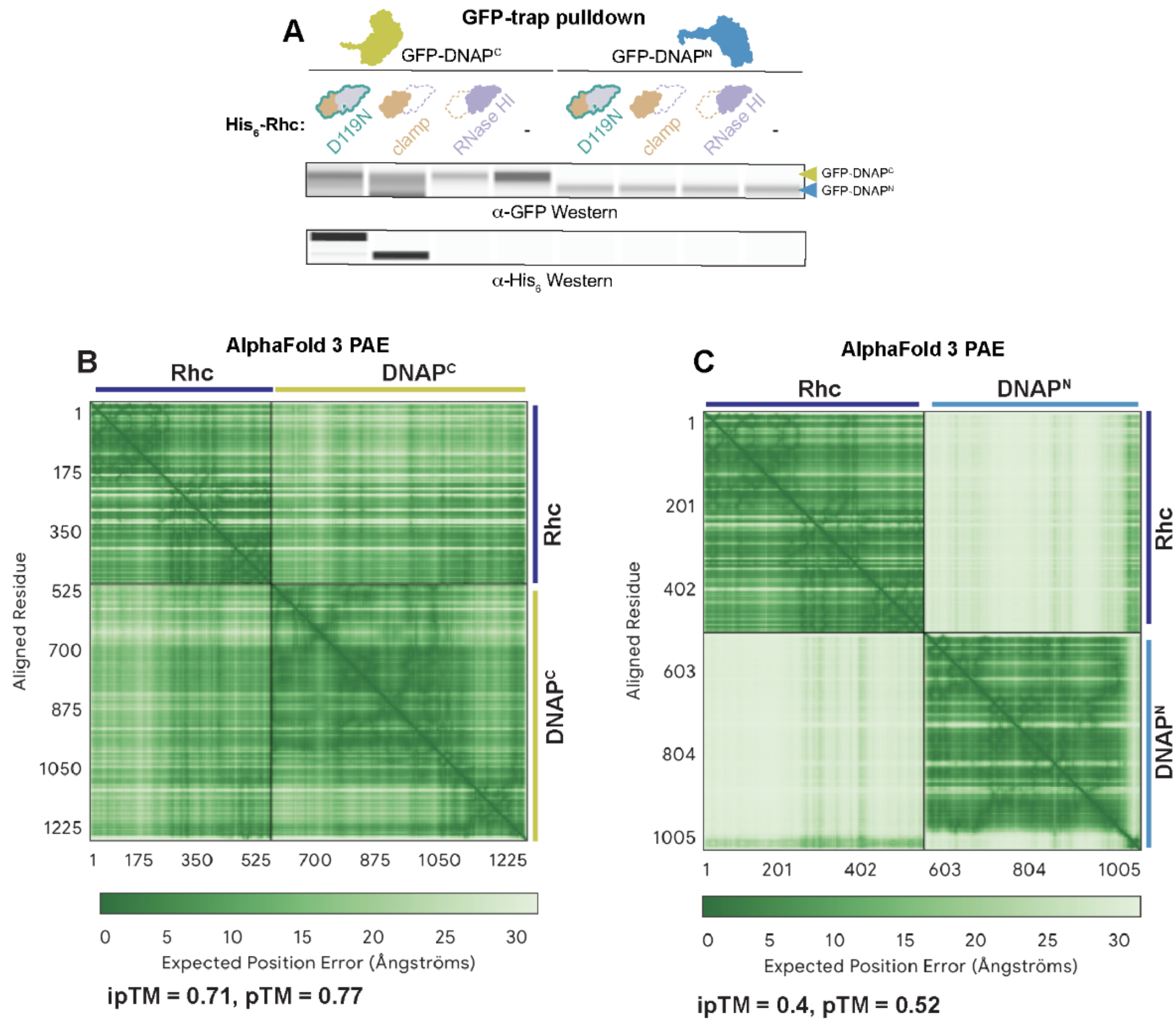

Figure S9. Rhc is a DNA polymerase subunit (A) GFP affinity pull-down assay examining interactions between phage DNA polymerase subunits (DNAP^C and DNAP^N) and Rhc. Elution fractions were visualized by Jess capillary western blotting against GFP (top) and His-6 (bottom). **(B)** PAE plot for the Rhc-DNAP^C complex. **(C)** PAE plot for the Rhc-DNAP^N complex.

bioRxiv preprint doi: https://doi.org/10.64898/2026.10.01.755736; this version posted October 2, 2026. The copyright holder for this preprint (which was not certified by peer review) is the author/funder, who has granted bioRxiv a license to display the preprint in perpetuity. It is made available under a CC-BY 4.0 International license.

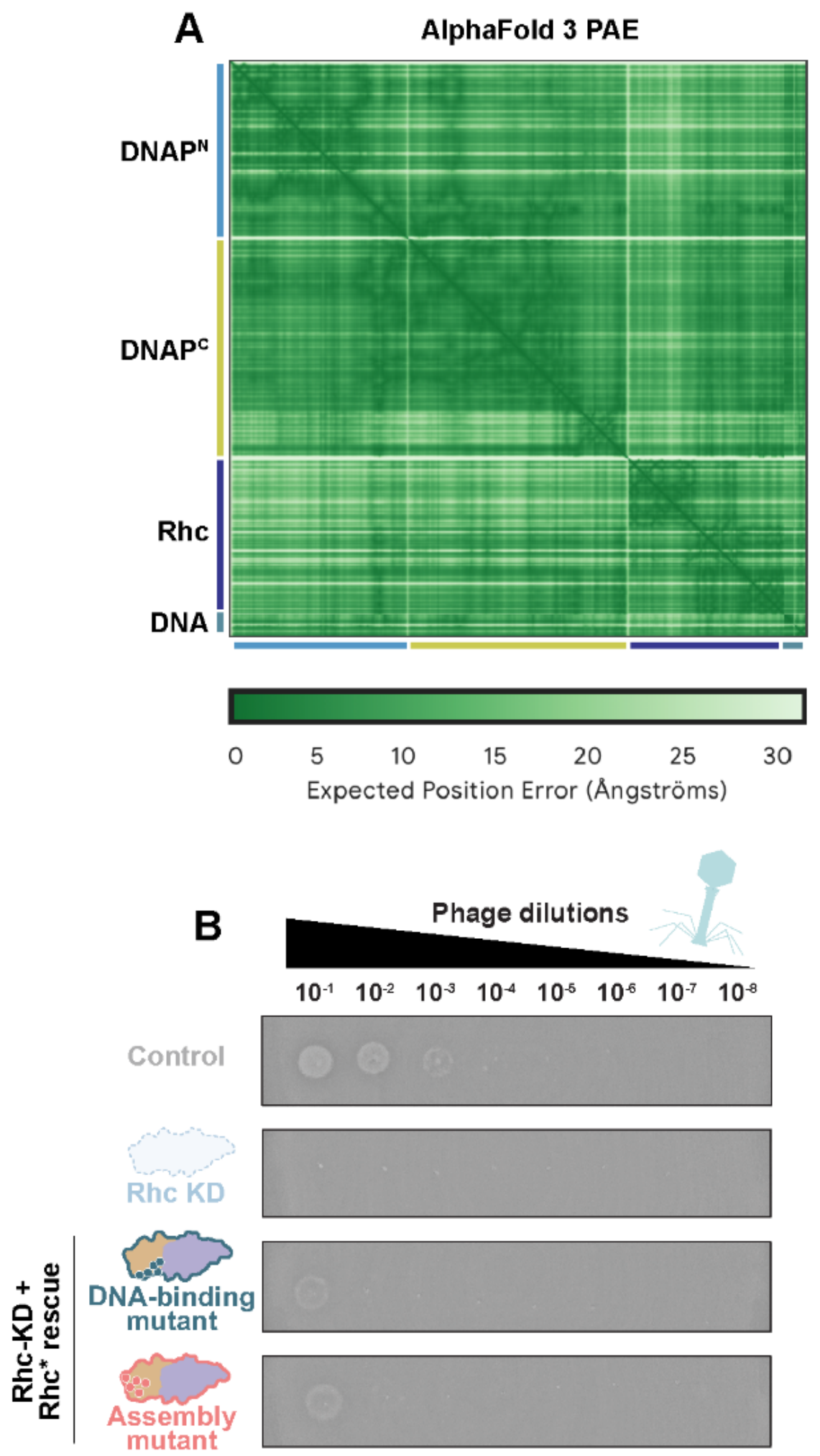

Figure S10. Rhc association with the DNA polymerase complex and requirement for productive infection. (A) AlphaFold3 predicted aligned error (PAE) plot for the complex comprising DNAP^N, DNAP^C, Rhc, and DNA. Lower PAE values indicate higher confidence in the relative positioning of the corresponding regions within the predicted complex. **(B)** Spot-titer assay showing productive Goslar infection under the indicated conditions. Serial 10-fold phage dilutions (10^{-1}-10^{-8}) were spotted on control cells, Rhc-knockdown (KD) cells, or Rhc-KD cells complemented with the indicated Rhc* DNA-binding or assembly mutants. Rhc depletion strongly impaired plaque formation, and neither mutant restored productive infection.

bioRxiv preprint doi: https://doi.org/10.64898/2026.10.01.755736; this version posted October 2, 2026. The copyright holder for this preprint (which was not certified by peer review) is the author/funder, who has granted bioRxiv a license to display the preprint in perpetuity. It is made available under a CC-BY 4.0 International license.

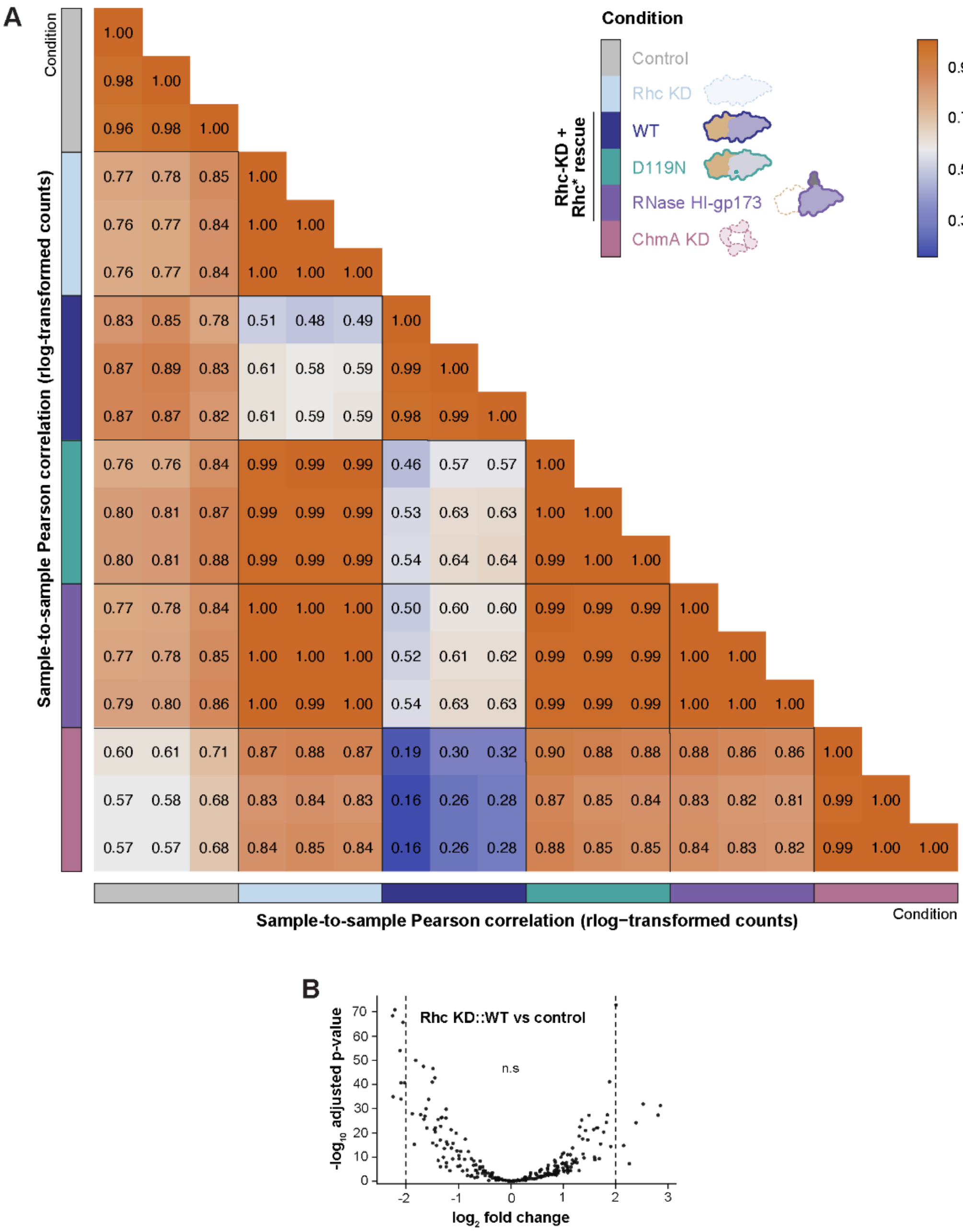

Figure S11. Sample-sample Pearson correlation analysis and Rhc complementation with WT Rhc. (A) Heatmap showing pairwise Pearson correlation coefficients calculated from rlog-transformed RNA-seq expression values across all samples, including non-targeting controls (Control), Rhc knockdown (Rhc KD), ChmA knockdown (ChmA KD), and RNaseH knockdown complemented with WT, RNase H catalytic mutant (D119N), or RNase H domain fused to gp173 (gp173 RNase H). Samples are hierarchically clustered based on correlation-derived distances, and correlation strength is indicated by the color scale (purple to yellow, low to high). Biological replicates cluster tightly within each condition, demonstrating high reproducibility. Full-length Rhc complementation clusters closely with non-targeting controls, whereas Rhc KD, catalytic mutant (D119N), and NTD-Taileboi conditions cluster separately, indicating that catalytic activity and full-length Rhc architecture are required to restore the global transcriptional state. Colored annotation bars indicate experimental conditions. **(B)** Volcano plot of RNA-seq differential expression analysis comparing Rhc knockdown (KD) complemented with wild-type (WT) Rhc with control. Each point represents a phage gene. The x-axis shows the $\log_2$ fold change, and the y-axis shows the $-\log_{10}$ adjusted *P* value. Dashed vertical lines indicate the fold-change threshold (±2), and the horizontal dashed line indicates the adjusted *P* value significance threshold (0.05). The majority of genes are not significantly differentially expressed, demonstrating that Rhc depletion does not broadly alter phage gene expression.

bioRxiv preprint doi: https://doi.org/10.64898/2026.10.01.755736; this version posted October 2, 2026. The copyright holder for this preprint (which was not certified by peer review) is the author/funder, who has granted bioRxiv a license to display the preprint in perpetuity. It is made available under a CC-BY 4.0 International license.

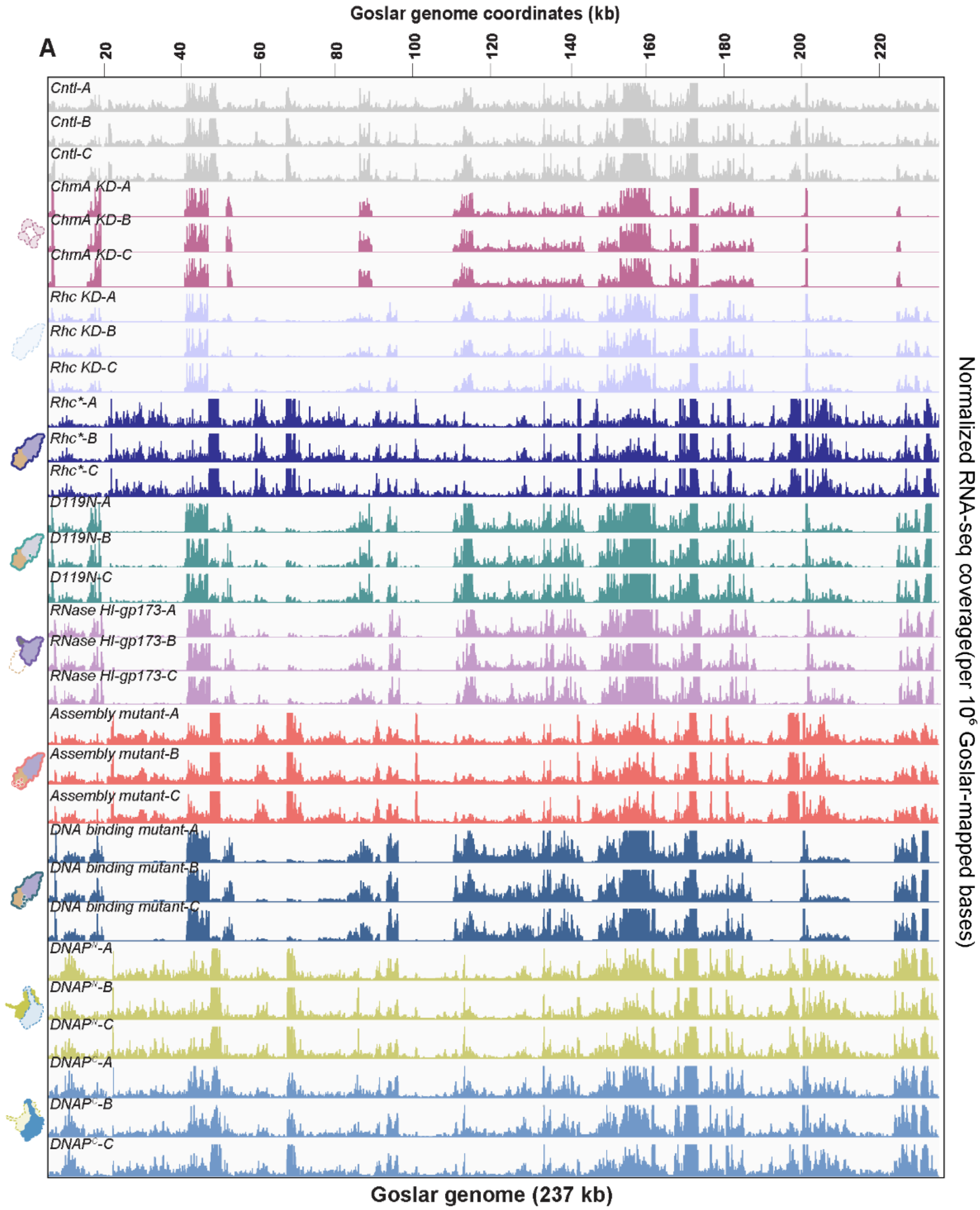

Figure S12. Transcriptomics profile of Rhc Knockdown and various complementations. Genome-wide RNA-seq coverage across the 237-kb Goslar genome under the indicated infection conditions. RNA-seq reads were aligned to a combined *E. coli*-Goslar reference, filtered to retain primary alignments with MAPQ ≥10 (mapping quality score), and reads mapping to the Goslar genome were extracted for visualization. For each sample, Goslar coverage was normalized by the total number of mapped Goslar bases using a scale factor of 10^6/(total Goslar-mapped bases), thereby accounting for differences in sequencing depth and read length between datasets. Normalized coverage tracks were exported as BigWig (.bw) files and visualized in IGV. Three biological replicates are shown for each condition, including the non-targeting control (Cntl), ChmA knockdown (ChmA KD), Rhc knockdown (Rhc KD), the catalytically inactive Rhc D119N mutant, RNase H I tagged to gp173 perturbation, DNA binding mutant,and depletion of the N- and C-terminal subunits of DNA polymerase subunits (DNAP^N and DNAP^C, respectively). IGV tracks were displayed at a fixed track height of 15. Goslar genome coordinates are shown above the tracks.

bioRxiv preprint doi: https://doi.org/10.64898/2026.10.01.755736; this version posted October 2, 2026. The copyright holder for this preprint (which was not certified by peer review) is the author/funder, who has granted bioRxiv a license to display the preprint in perpetuity. It is made available under a CC-BY 4.0 International license.

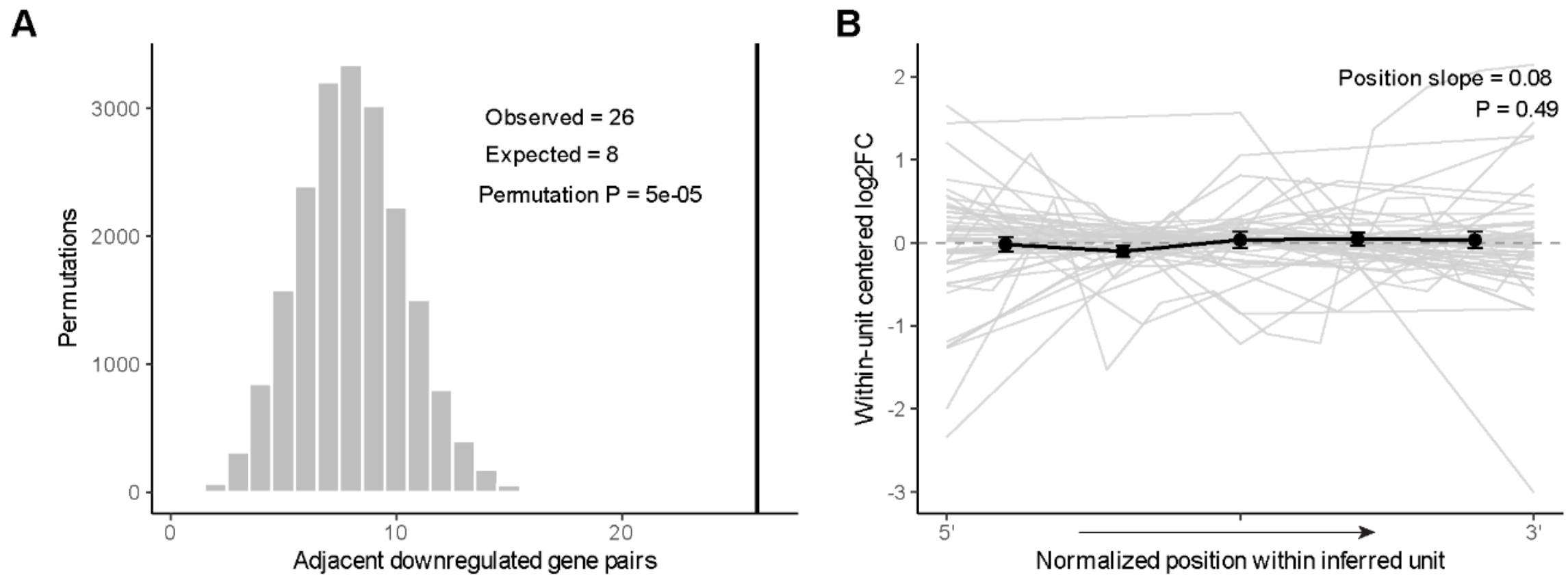

Figure S13. Rhc-dependent transcriptional defects are non-randomly clustered but do not show progressive 5′ to 3′ loss within putative transcriptional units. (A) Permutation analysis of genomic clustering of Rhc-dependent genes. Rhc-dependent downregulated genes were defined as genes with adjusted $P < 0.05$ and $\log_2$ fold change ≤ -2 in Rhc knockdown relative to control. The observed number of adjacent downregulated gene pairs was compared with the distribution obtained from 20,000 random permutations while preserving the number of downregulated genes on each strand. The observed dataset contained 26 adjacent downregulated gene pairs, compared with a random expectation of approximately 8 pairs. The vertical line indicates the observed value. The observed clustering exceeded that obtained in the randomized datasets (empirical permutation $P = 5 \times 10^{-5}$), demonstrating that Rhc-dependent downregulated genes are significantly more spatially clustered than expected by chance. **(B)** Analysis of transcriptional position within inferred transcriptional units. Inferred units were defined as contiguous genes on the same strand separated by ≤100 bp. Genes within each unit were ordered according to their true 5′ to 3′ transcriptional direction, with minus-strand units reversed relative to genomic coordinate order. Gene position within each unit was normalized from 0 at the 5′ end to 1 at the 3′ end, allowing transcriptional units of different sizes to be compared on a common scale. To isolate within-unit positional effects from differences in overall expression between units, the mean $\log_2$ fold change of each unit was subtracted from the $\log_2$ fold change of each constituent gene, generating a within-unit centered $\log_2$ fold change. Gray lines represent individual inferred transcriptional units, and black points and lines summarize the mean positional trend. Linear regression of centered $\log_2$ fold change against normalized 5′-to-3′ position yielded a slope of 0.08 ($P = 0.49$), indicating no significant progressive increase in downregulation toward the 3′ ends of inferred transcriptional units. Thus, although Rhc-dependent genes are strongly clustered across the genome, the transcriptional defect is not consistent with a simple genome-wide model of progressively reduced RNA polymerase processivity from 5′ to 3′.

bioRxiv preprint doi: https://doi.org/10.64898/2026.10.01.755736; this version posted October 2, 2026. The copyright holder for this preprint (which was not certified by peer review) is the author/funder, who has granted bioRxiv a license to display the preprint in perpetuity. It is made available under a CC-BY 4.0 International license.

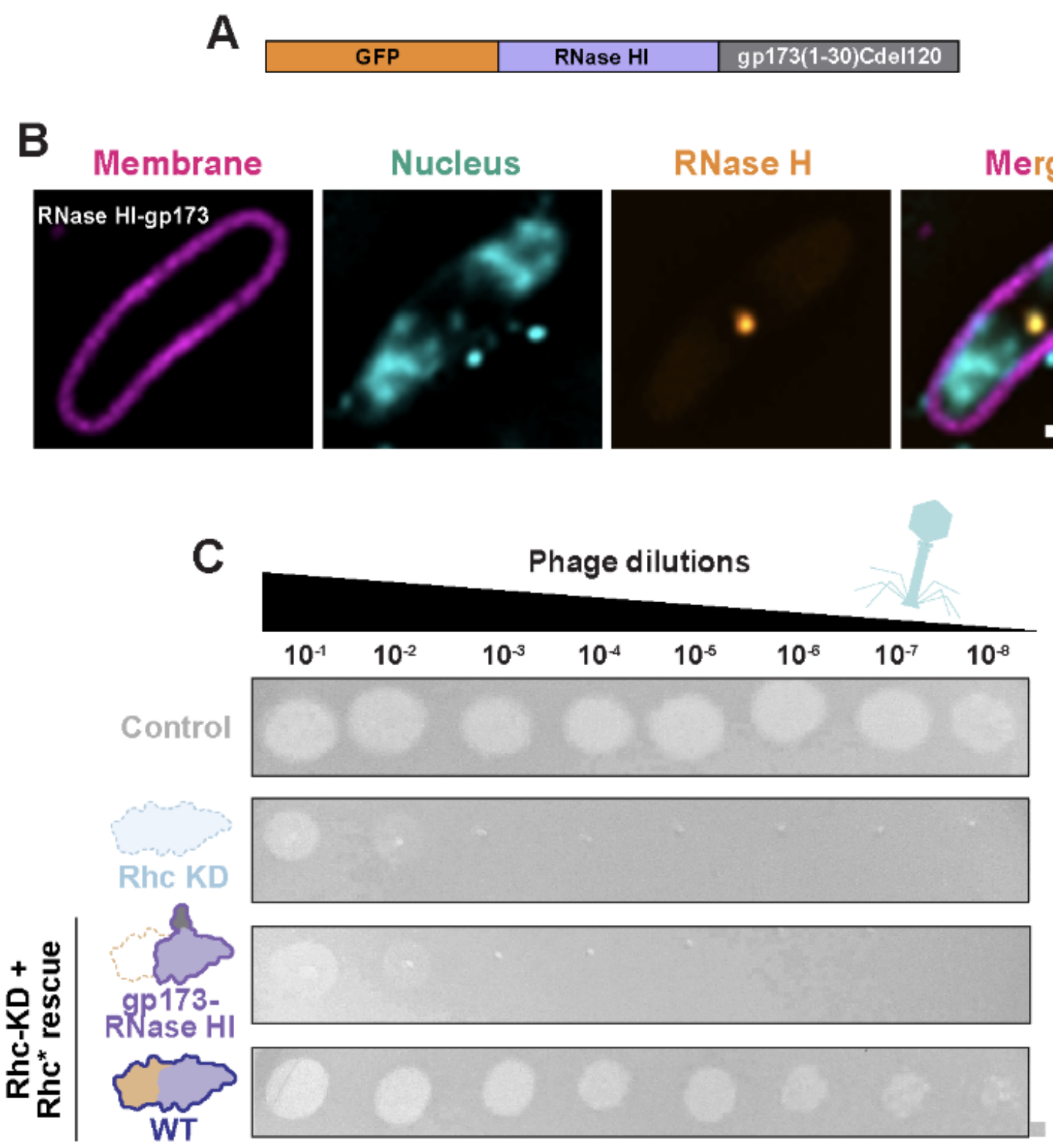

Figure S14. Forced nuclear targeting of the N-terminal RNase H domain is insufficient to rescue phage replication. (A) Schematic of the fusion construct used to force nuclear targeting of the RNaseH N-terminal domain (NTD). GFP-tagged RNaseH-NTD was fused to residues 1-30 of gp173 (Taileboi), with deletion of residues 1-120, a construct previously shown to localize to the phage nucleus. **(B)** Fluorescence microscopy showing forced localization of the N-terminal RNase H domain to the nascent nucleus. **(C)** Spot titer assay showing that forced nuclear localization of the N-terminal RNase H domain fails to rescue phage propagation and lysis.

bioRxiv preprint doi: https://doi.org/10.64898/2026.10.01.755736; this version posted October 2, 2026. The copyright holder for this preprint (which was not certified by peer review) is the author/funder, who has granted bioRxiv a license to display the preprint in perpetuity. It is made available under a CC-BY 4.0 International license.

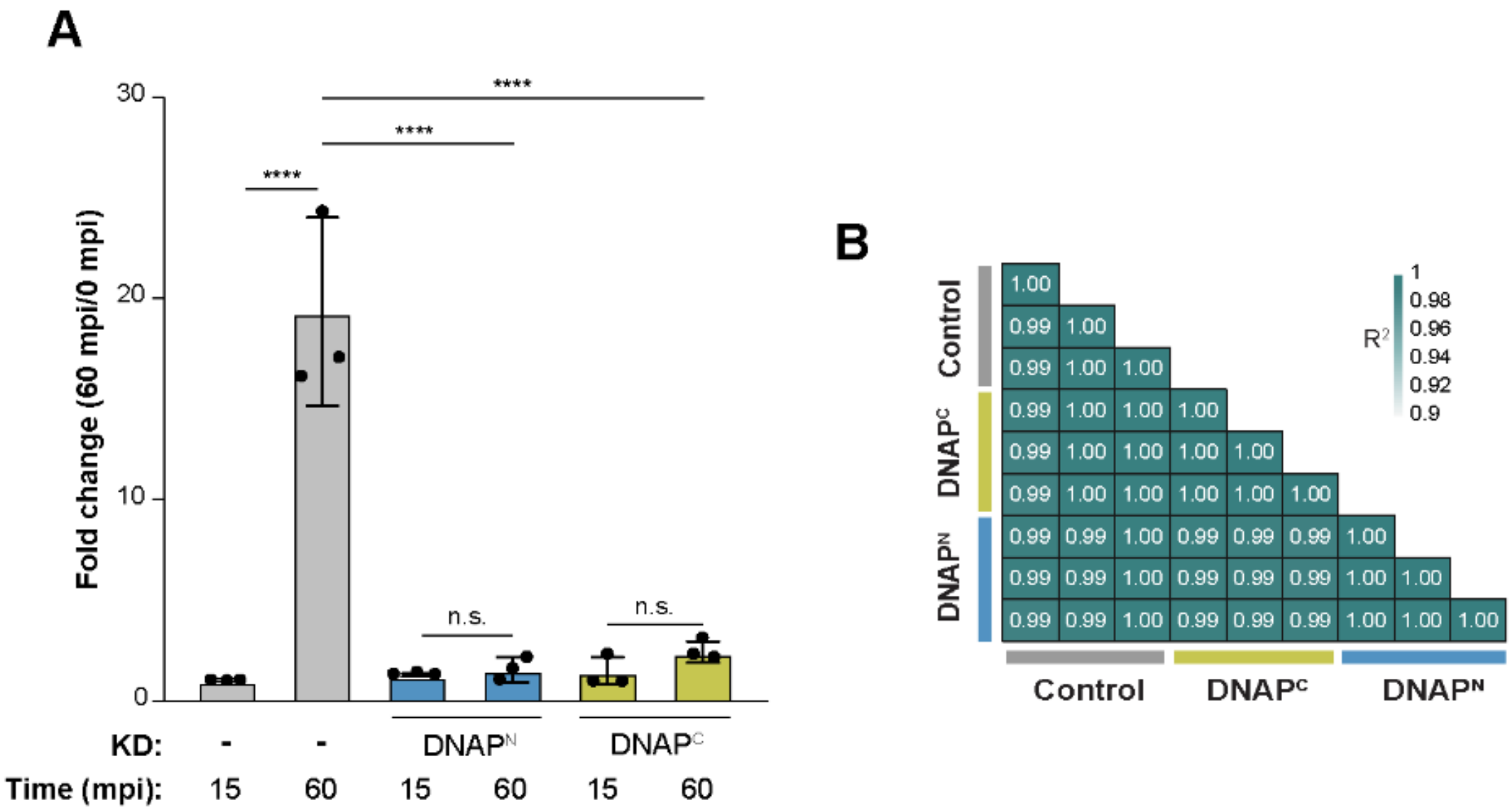

Figure S15. DNA polymerase knockdown disrupts phage genome replication while largely preserving the transcriptional program. (A). Measured at indicated time points and normalized by 0 mpi. Control infections show robust genome amplification at 60 mpi, whereas DNAP knockdowns (DNAP^N and DNAP^C KD) infections fail to support DNA replication. Bars represent mean ± s.e.m. (n = 3 biological replicates). Statistical significance was assessed by one-way ANOVA followed by Šídák's multiple comparisons test (α = 0.05). Comparisons of control versus KDs conditions were highly significant (****$P < 0.0001$). **(B).** Pairwise correlation matrix comparing genome-wide phage RNA-seq profiles from the non-targeting control, DNAP^C-depleted, and DNAP^N-depleted infections. Individual tiles represent pairwise coefficients of determination, R-squared. The high correlations among conditions indicate that depletion of either DNA polymerase subunit has little effect on the overall phage transcriptional profile.

bioRxiv preprint doi: https://doi.org/10.64898/2026.10.01.755736; this version posted October 2, 2026. The copyright holder for this preprint (which was not certified by peer review) is the author/funder, who has granted bioRxiv a license to display the preprint in perpetuity. It is made available under a CC-BY 4.0 International license.

SUPPLEMENTAL TABLES

Table S1. Goslar Rhc C-terminal domain DALI hits Top 25 hits from the PDB25 subset. Light green highlight indicates sliding clamp proteins

PDB-Chain	Z score	Calpha rmsd	Alignment length	Uniprot ID	Description
3ggr-B	7.1	4.7	179	O60921	H. sapiens HUS1
7st9-G	5.9	4	166	Q08949	S. cerevisiae Ddc1
3a1j-A	5.7	4	156	Q99638	H. sapiens RAD9A
7st9-H	5.3	4.8	180	Q02574	S. cerevisiae Mec3
7ype-A	5.3	4.3	147	A0A0A1E3R5	African swine fever virus E301R
2awa-B	4.9	4.5	159	O06672	Streptococcus pneumomiae TIGR4 (DNA Pol III beta chain)
3hi8-A	4.7	4.5	148	D0VWY8	Haloferax volcanii PCNA
6drt-A	4.7	3.8	143	P04525	phage T4 gp45
4ftx-A	4.5	3.8	87	P38247	S. cerevisiae Slm4
3ggr-C	4.4	3.6	148	O60671	H. sapiens RAD1
1tu1-A	4.3	4.9	84	Q9I738	P. aeruginosa PA94
6y2p-A	4.3	5.4	71	P52129	E. coli RnlA toxin
7wmp-a	4.3	3.2	75	I7HHN4	Helicobacter phage KHP30 portal
6e8a-A	4.2	3.9	81	Q7CPJ3	Salmonella enterica DcrB
5xqi-B	4.2	4.8	51	Q9GZN7	H. sapiens Rogdi
7kzn-I	4.2	3.9	67	Q9SWQ6	C. reinhardtii dynein light chain roadblock LC7a
6aig-A	4.2	5.5	154	Q9YFT8	Aeropyrum pernix K1 PCNA
2du7-A	4	6	97	Q59054	M. jannaschii O-phosphoseryl-tRNA synthetase
8yqg-B	4	3.2	70	A0A5B9DBT7	Promethearchaeum syntrophicum longin domain protein
8es4-E	4	11.7	67	A0A482JLu9	Shigella phage Buco gp40
6n90-A	4	3.4	70	A9CID9	Agrobacterium tumefaciens YoeB
4kc5-A	3.9	7.9	114	A1KQS1	Mycetohabitans rhizoxinica RhiE
6e8e-A	3.8	4.5	156	P0A988	E. coli beta sliding clamp DnaN
6xrb-A	3.8	7.5	92	A0A0H3NHS6	Salmonella enterica SciW
5iwd-A	3.8	4.3	134	P16790	Human cytomegalovirus UL44

Table S2. Goslar GFP-Rhc IP-MS results Pink shading indicates an RNA polymerase subunit; green shading indicates a DNA polymerase subunit.

NCBI #	Name	Sequence coverage (%)	# peptides	Found in GFP control?
YP_009820929.1	putative ribonuclease HI HOV27_gp190	81	77	No
YP_009820859.1	Nuclear import factor PicA HOV27_gp174	25	9	Yes
YP_009820893.1	hypothetical protein HOV27_gp226	30	5	Yes

bioRxiv preprint doi: https://doi.org/10.64898/2026.10.01.755736; this version posted October 2, 2026. The copyright holder for this preprint (which was not certified by peer review) is the author/funder, who has granted bioRxiv a license to display the preprint in perpetuity. It is made available under a CC-BY 4.0 International license.

NCBI #	Name	Sequence coverage (%)	# peptides	Found in GFP control?
YP_009820873.1	Nuclear shell protein ChmA HOV27_gp246	26	10	Yes
YP_009820726.1	Major capsid protein HOV27_gp041	11	6	Yes
YP_009820905.1	hypothetical protein HOV27_gp214	46	5	Yes
YP_009820723.1	hypothetical protein HOV27_gp038	8	5	Yes
YP_009820856.1	nvRNAP subunit HOV27_gp171	9	4	Yes
YP_009820753.1	DNA Polymerase HOV27_gp068	8	3	No
YP_009820708.1	hypothetical protein HOV27_gp023	25	2	No
YP_009820724.1	nvRNAP subunit HOV27_gp039	7	2	No
YP_009820755.1	hypothetical protein HOV27_gp070	22	2	Yes
YP_009820782.1	hypothetical protein HOV27_gp097	35	2	No
YP_009820861.1	Nuclear shell-associated protein ChmC HOV27_gp176	13	2	No
YP_009820743.1	hypothetical protein HOV27_gp058	7	2	Yes
YP_009820689.1	hypothetical protein HOV27_gp004	14	2	Yes
YP_009820704.1	hypothetical protein HOV27_gp019	16	1	Yes
YP_009820788.1	hypothetical protein HOV27_gp103	17	1	Yes
YP_009820850.1	nvRNAP subunit HOV27_gp165	2	1	No
YP_009820869.1	hypothetical protein HOV27_gp184	8	1	No
YP_009820882.1	hypothetical protein HOV27_gp237	4	1	No
YP_009820794.1	hypothetical protein HOV27_gp109	9	1	No
YP_009820746.1	hypothetical protein HOV27_gp061	4	1	No
YP_009820789.1	hypothetical protein HOV27_gp104	17	1	Yes
YP_009820694.1	Putative prohead protease HOV27_gp009	4	1	No
YP_009820756.1	hypothetical protein HOV27_gp071	9	1	No
YP_009820931.1	hypothetical protein HOV27_gp188	3	1	No
YP_009820860.1	nvRNAP subunit HOV27_gp175	2	1	No
YP_009820811.1	hypothetical protein HOV27_gp126	2	1	No
YP_009820823.1	hypothetical protein HOV27_gp138	16	1	Yes
YP_009820766.1	hypothetical protein HOV27_gp081	2	1	No
YP_009820875.1	DNA Polymerase HOV27_gp244	2	1	No
YP_009820762.1	hypothetical protein HOV27_gp077	5	1	No

bioRxiv preprint doi: https://doi.org/10.64898/2026.10.01.755736; this version posted October 2, 2026. The copyright holder for this preprint (which was not certified by peer review) is the author/funder, who has granted bioRxiv a license to display the preprint in perpetuity. It is made available under a CC-BY 4.0 International license.

Table S3: *E. coli MC1000 and Rossetta* strains were used in this study. GFP is "sfGFP" which is Addgene Plasmid #48885 pTD103luxI_GFP with mutations S2A, T65G, D190H and D197H. Plasmids with p15A-CmR backbone: PR26_Goslar_gp190KD_G2. Plasmids with pDSW206 backbone: PR39, PR24, PR41, PR60, PR47, PR67, PR68, and Plasmids with 2BT backbone are: PR27, PR28, PR29, PR61, PR70, PR73, PR74, PR75, P76.

S.N.	Strain name	Plasmid name	Expressed Protein/Guide
1	PR26	pPR26_Goslar_gp190KD_G2	dRfxCas13d+guideRNA-Rhc (tgccattgtaaacctcattaatcatt-gtccg)
2	PR26::PR39	pPR39_Goslar_gp190_Nter-GFP	dRfxCas13d+guideRNA+Full length Rhc
3	PR26::PR24	pPR24_Goslar_gp190_Nter-GFP_CterRFp_deltaCTD	dRfxCas13d+guideRNA+RNase HI (NTD)
4	PR26::PR41	pPR41_Gos-lar_gp190_Cter_NterGFP	dRfxCas13d+guideRNA+Rhc Clamp
5	PR26::PR67	pPR72_DNA_nonbind-ing_GFP_RNaseH	dRfxCas13d+guideRNA+Full length Rhc where R318, R233, K259, K262, R264 mutated to A
6	PR26::PR68	pPR68_DNAP non-bind-ing_GFP_RNaseH	dRfxCas13d+guideRNA+Full length Rhc with following mutations- S272D, L363D, W365A, Y505E, L507A
9	PR26::PR60	pr60_goslar_gp190_nter-gfp_cter-codalt1-30_gp173cdel120ter_deltactd	dRfxCas13d+guideRNA+RNase HI+delta-CTD-gp173
10	PR26::PR47	pr47_goslar_gp190_Nter-GFP_Asp119Asn	dRfxCas13d+guideRNA+D119N mutated Rhc
11	PR61 in Rossetta	pPR61_D119N_2BT_His_FL_RNaseH	D119N mutated Rhc for purification
12	PR27 in Rossetta	pPR27_2BT-Tev-His_gp190	Rhc purification
13	PR28 in Rossetta	pPR28_2BT-Tev-His_gp190_N-ter	RNase HI domain purification
14	PR29 in Rossetta	pPR29_2BT-Tev-His_gp190_C-ter	Clamp purification
15	PR70 in Rossetta	pPR70_2BT-Tev-His_DNA_binding_mutant	DNA binding mutant purification
16	PR73 in Rossetta	PR73_gp244	DNAP C-subunit purification
17	PR74 in Rossetta	PR74_N-ter-His_gp068	DNAP N-subunit purification
18	PR75 in Rossetta	PR75_N-ter-His_Assem-bly_mutant	Rhc Assembly mutant purification
19	MN008	pBA562_ddCasRX_Goslar-HOV27_gp177-g1	dRfxCas13d+guideRNA1-gp177(aacgtgcgggcgtacata-tcgctcatacccc)
20	MN018	pBA562_ddCasRX_Goslar-HOV27_gp177-g2	dRfxCas13d+guideRNA2-gp177(taatcacctaacgtgcgggcg-tacatatcgc)

bioRxiv preprint doi: https://doi.org/10.64898/2026.10.01.755736; this version posted October 2, 2026. The copyright holder for this preprint (which was not certified by peer review) is the author/funder, who has granted bioRxiv a license to display the preprint in perpetuity. It is made available under a CC-BY 4.0 International license.

Table S4. qPCR primers used in this study.

Primer name	Sequence	Source
GosChmAqPCR_F10	TACGCTGTACTGGTTCGGG	IDT
GosChmAqPCR_R10	GGTACTGAATCTCCGTCGTCTG	IDT

Table S5. DESeq2 analysis of knockdown conditions compared with the control.

Table S6. RNA-seq quality control metrics and sequencing coverage across all samples.

Table S7. Comparative differential expression analysis of Rhc knockdown and ChmA knockdown relative to control.

Table S8. Subset of RNA-seq differential expression results for Rhc KD versus NT control, filtered from Table S5.

Gene ID	Base mean	log2 fold change	Adjusted P value	Status
HOV27_gp246	58833.7	2.95	1.98E-106	Upregulated
HOV27_gp214	13458.5	2.63	4.55E-81	Upregulated
HOV27_gp176	7210.9	2.10	9.11E-46	Upregulated
HOV27_gp144	4451.2	2.04	3.92E-52	Upregulated
HOV27_gp022	435.3	-7.43	7.32E-106	Downregulated
HOV27_gp023	661.5	-6.79	2.79E-171	Downregulated
HOV27_gp217	1548.2	-4.15	4.25E-56	Downregulated
HOV27_gp093	289.0	-4.01	2.73E-55	Downregulated
HOV27_gp091	197.2	-4.00	1.78E-24	Downregulated
HOV27_gp056	599.3	-3.72	4.01E-65	Downregulated
HOV27_gp057	662.4	-3.62	3.41E-36	Downregulated
HOV27_gp203	841.8	-3.62	1.45E-42	Downregulated
HOV27_gp013	306.7	-3.61	6.86E-30	Downregulated
HOV27_gp024	450.5	-3.57	8.24E-60	Downregulated
HOV27_gp202	424.2	-3.54	2.64E-45	Downregulated
HOV27_gp092	1751.6	-3.50	2.42E-51	Downregulated
HOV27_gp076	276.2	-3.43	2.11E-36	Downregulated
HOV27_gp055	631.4	-3.40	6.98E-56	Downregulated
HOV27_gp079	388.5	-3.28	5.77E-34	Downregulated
HOV27_gp218	5635.3	-3.28	2.51E-34	Downregulated
HOV27_gp028	516.2	-3.24	2.06E-51	Downregulated
HOV27_gp030	696.6	-3.23	1.42E-37	Downregulated
HOV27_gp025	1827.2	-3.23	1.6E-36	Downregulated

bioRxiv preprint doi: https://doi.org/10.64898/2026.10.01.755736; this version posted October 2, 2026. The copyright holder for this preprint (which was not certified by peer review) is the author/funder, who has granted bioRxiv a license to display the preprint in perpetuity. It is made available under a CC-BY 4.0 International license.

Gene ID	Base mean	log2 fold change	Adjusted P value	Status
HOV27_gp192	316.1	-3.22	2.11E-36	Downregulated
HOV27_gp204	3739.9	-3.21	1.24E-36	Downregulated
HOV27_gp026	2049.3	-3.14	1.94E-37	Downregulated
HOV27_gp027	623.0	-3.11	7.18E-40	Downregulated
HOV27_gp029	2272.4	-3.10	4.88E-46	Downregulated
HOV27_gp036	1517.1	-3.03	8.42E-44	Downregulated
HOV27_gp063	1710.3	-3.03	7.48E-29	Downregulated
HOV27_gp224	473.0	-3.01	6.72E-41	Downregulated
HOV27_gp062	603.2	-2.95	4.9E-28	Downregulated
HOV27_gp052	131.4	-2.93	4.22E-17	Downregulated
HOV27_gp090	1828.3	-2.93	6.45E-31	Downregulated
HOV27_gp189	527.0	-2.92	1.16E-41	Downregulated
HOV27_gp035	1350.0	-2.90	2.36E-44	Downregulated
HOV27_gp161	1926.1	-2.74	1.76E-58	Downregulated
HOV27_gp084	263.7	-2.73	3.95E-21	Downregulated
HOV27_gp033	891.5	-2.71	1.93E-27	Downregulated
HOV27_gp160	929.2	-2.70	7.74E-32	Downregulated
HOV27_gp219	658.3	-2.68	1.09E-36	Downregulated
HOV27_gp188	1018.6	-2.67	8.1E-34	Downregulated
HOV27_gp031	986.2	-2.63	3.43E-32	Downregulated
HOV27_gp086	1376.8	-2.62	8.01E-30	Downregulated
HOV27_gp205	756.0	-2.61	8.14E-30	Downregulated
HOV27_gp048	753.8	-2.61	2.15E-39	Downregulated
HOV27_gp061	1305.2	-2.56	1.06E-30	Downregulated
HOV27_gp032	977.9	-2.55	4.26E-31	Downregulated
HOV27_gp085	409.4	-2.54	1.84E-26	Downregulated
HOV27_gp087	366.0	-2.54	1.66E-22	Downregulated
HOV27_gp034	1208.2	-2.53	4.05E-33	Downregulated
HOV27_gp049	468.5	-2.50	1.01E-29	Downregulated
HOV27_gp187	461.0	-2.43	6.42E-19	Downregulated

bioRxiv preprint doi: https://doi.org/10.64898/2026.10.01.755736; this version posted October 2, 2026. The copyright holder for this preprint (which was not certified by peer review) is the author/funder, who has granted bioRxiv a license to display the preprint in perpetuity. It is made available under a CC-BY 4.0 International license.

Gene ID	Base mean	log2 fold change	Adjusted P value	Status
HOV27_gp041	11605.9	-2.28	4.56E-23	Downregulated
HOV27_gp054	727.7	-2.25	3.78E-28	Downregulated
HOV27_gp088	1032.8	-2.24	1.73E-14	Downregulated
HOV27_gp051	873.4	-2.13	6.2E-20	Downregulated
HOV27_gp220	1725.4	-2.12	1.01E-38	Downregulated
HOV27_gp089	499.8	-2.07	6.26E-20	Downregulated
HOV27_gp060	1719.5	-2.04	2.57E-17	Downregulated
HOV27_gp058	6611.3	-2.00	2.51E-21	Downregulated

Table S9. Rhc_dependent_transcriptional_blocks. Rhc_B1-Rhc_B16 denote 16 putative discrete genomic blocks of coordinately downregulated, adjacent genes following Rhc depletion. Block size indicates the number of genes within each block (2-5 genes), and position in block indicates gene order in the 5′→3′ transcriptional direction. Block start/end and length define the genomic coordinates and span of each affected region. baseMean represents mean normalized expression, log2FoldChange the expression change upon Rhc depletion, and pvalue/padj the statistical significance before and after multiple-testing correction, respectively.

Table S10. RNA-seq Rhc KD::WT* vs NT control

Gene ID	Base mean	log2 fold change	Adjusted P value	Status
HOV27_gp059	1813.0	2.85	5.24E-32	Upregulated
HOV27_gp042	1676.9	2.81	4.53E-28	Upregulated
HOV27_gp058	6611.3	2.52	1.25E-32	Upregulated
HOV27_gp041	11605.9	2.38	7.38E-25	Upregulated
HOV27_gp190	17902.7	2.26	6.48E-08	Upregulated
HOV27_gp218	5635.3	2.15	1.81E-15	Upregulated
HOV27_gp157	3445.7	2.01	1.28E-73	Upregulated
HOV27_gp182	1902.5	-2.26	3.56E-69	Downregulated
HOV27_gp156	675.2	-2.25	1.18E-35	Downregulated
HOV27_gp183	2223.3	-2.21	1.24E-71	Downregulated
HOV27_gp071	1775.2	-2.12	9.36E-55	Downregulated
HOV27_gp227	696.3	-2.10	1.2E-34	Downregulated
HOV27_gp075	1231.1	-2.10	2.15E-41	Downregulated
HOV27_gp070	1955.0	-2.06	1.99E-66	Downregulated
HOV27_gp225	1781.1	-2.03	2.32E-41	Downregulated
